# Increased number and activation of peripheral basophils in adult‐onset minimal change disease

**DOI:** 10.1111/jcmm.15417

**Published:** 2020-06-08

**Authors:** Huanqin Han, Yong‐Zhi Xu, Shuzhen Liao, Haiyan Xiao, Xiaoqun Chen, Xing Lu, Shujun Wang, Chen Yang, Hua‐feng Liu, Qingjun Pan

**Affiliations:** ^1^ Infectious Diseases Center Affiliated Hospital of Guangdong Medical University Zhanjiang China; ^2^ Key Laboratory of Prevention and Management of Chronic Kidney Disease of Zhanjiang City Institute of Nephrology Affiliated Hospital of Guangdong Medical University Zhanjiang China; ^3^ College of Nursing Department of Anesthesiology and Perioperative Medicine Augusta University Augusta GA USA

**Keywords:** activation, adult, basophil, minimal change disease, recurrence

## Abstract

Nowadays, the pathogenesis of minimal change disease (MCD) is still not well‐known, and the current understanding on MCD is mainly based on data derived from children, and very few adults. Here, we comprehensively analysed the correlation between the changes of peripheral basophils and the incidence rate and relapse of adult‐onset MCD. The results showed that in patients at the onset of MCD, the ratio and activation of basophils were all higher than those of healthy controls (all *P* < .05). In vitro test results showed that basophils from healthy controls can be activated by the serum taken from patients with MCD. Among 62 patients at the onset of MCD, with complete remission after treatment and 1 year of follow‐up, the relative and absolute basophil counts before treatment were higher in the long‐term remission group (n = 33) than that of the relapse group (n = 29). The basophil counts were significantly higher in the infrequent relapse group (n = 13) than that of the frequent relapse group (n = 16; *P* < .05). These findings suggested that basophil may play a pathogenic role in adult‐onset MCD, and the increased number and activation of peripheral basophils could predict recurrence in adult MCD.

## 1 INTRODUCTION

Minimal change disease (MCD) is a combination of glomerular diseases that presents with nephrotic syndrome. The cells show no obvious pathological changes under the light microscope, but extensive elimination of podocytic processes can be observed with an electron microscope. MCD is a pathological diagnosis, accounting for 70%‐90% of primary nephrotic syndrome in children and 10%‐15% of nephrotic syndrome in adults.^1^

The current understanding on MCD is mainly based on data derived from children and very few adults. In most patients, MCD is responsive to glucocorticoid therapy and can be easily treated; however, there is an extremely high relapse rate.^2^ Moreover, MCD may transform into even focal segmental glomerulonephritis and end‐stage renal disease.^3^, ^4^ The pathogenic mechanisms of MCD are currently unclear. Some studies have indicated that T lymphocytes, particularly T helper (Th) cells, affect the onset of MCD.^5^, ^6^ After Th cells differentiate into Th2 cells, the ones expressing high levels of IL‐4 and IL‐13 can participate in the onset of MCD.^7^, ^8^, ^9^, ^10^, ^11^, ^12^ Importantly, patients with MCD have low serum IgG levels^13^ and elevated IgE levels,^14^, ^15^ suggesting that humoural immunity is involved in the onset of MCD. The onset of MCD is related to circulating immune factors^16^ and proteinuria induced by podocyte dysfunction and podocyte injury^17^; however, other mechanisms may also be involved in the pathogenesis of MCD, and the pathogenic factors involved in these mechanisms have not yet been identified. A single theory cannot fully explain the pathophysiological course of MCD, and more details on the mechanisms involved in this process are needed.

Pirotzky et al^18^ found that patients with MCD showed sensitization and basophils might participate in pathological events in MCD. Additionally, Mack et al^19^ suggested that basophils ‘should be analysed in minimal change disease and focal segmental glomerulonephritis’. However, the effects of basophils on MCD have not been further investigated. In recent years, as techniques for detecting basophils have become more advanced, researchers have demonstrated that basophils have more important functions in allergic reactions and immune regulation.^20^

In this study, by detecting the quantity and activation of peripheral basophils in adult patients with MCD, we comprehensively analysed the relationship between basophils, MCD onset and MCD relapse for the first time. We also examined the effect of the serum from adult patients with MCD on the activation of basophils from normal individuals in vitro.

## 2 MATERIALS AND METHODS

### 2.1 Patients and controls

This study included patients at the initial onset of MCD, who were admitted to the Affiliated Hospital of Guangdong Medical University from January 2015 to February 2018. All patients were adults, and were diagnosed with idiopathic MCD, and met the diagnostic criteria of nephrotic syndrome.^21^ The control groups were healthy physical examinees whose gender and age were matched with the experimental groups.

### 2.2 Therapeutic regimen

Oral prednisone 1 mg/kg/d or an equal dose of methylprednisolone was used as an original dose. Then it was reduced by about 10% of the original dose every 2 weeks. Until the dose of prednisone was 10 mg/d, it was maintained for about 6 months and then the drug was slowly withdrawn. The total therapeutic course was 9‐12 months. In patients with steroid resistance or frequent relapse, it might be combined with an immunosuppressant, such as cyclophosphamide.

### 2.3 Definitions

Relapses were defined as follows: (a) relapse, relapse after urine protein was negative with urine protein ≥1+; (b) frequent relapse, more than two episodes of relapse in 6 months or more than three episodes of relapse in 1 year; (c) long‐term remission, no recurrence after urine protein was negative for more than 1 year.^22^, ^23^

### 2.4 Follow‐up procedure in patients with MCD

Cases from 2015 to 2016 were retrospectively analysed for different relapse outcomes after the treatment and differences in the counts and proportions of basophils before treatment (as determined by routine blood tests). There were 85 patients at the initial onset of MCD, who had follow‐ups until December 2017; 62 patients, who had follow‐ups for 1 year after their urinary protein was negative after initial treatment; 33 patients with long‐term remission; and 29 patients with relapse (including 16 cases of frequent relapse and 13 cases of infrequent relapse).

### 2.5 Experimental method and flow cytometry analysis

Peripheral venous blood samples were collected from patients at the initial onset of MCD, beginning in 2017, who had received no glucocorticoids or immunosuppressants. The samples were processed within 2 hours, and flow cytometry was used to determine basophil (CD123^+^CD203c^+^) ratios, activation indexes (mean fluorescence intensity [MFI] of CD203c and CD62L), and intracellular factors expressed by basophils (IL‐4, IL‐6 and IL‐13). In total, 17 patients were included, with a mean age of 27.7 ± 13.3 years. During the same period, healthy volunteers were enrolled as the normal control group, including 22 individuals with a mean age of 26.1 ± 2.0 years.

The peripheral blood samples were divided into extracellular and intracellular tubes. In the extracellular tube, human basophils were gated on CD203c‐PE and CD123‐Alexa Fluor 647 (BioLegend) double‐positive cells after extracellular staining. MFI of CD203c‐PE and CD62L‐PE‐Cy7 (BioLegend) expression was quantified as the activation indexes of basophils. In the intracellular tube, human basophils were gated on CD203c‐PE‐Cy7 (eBioscience) and CD123‐Alexa Fluor 647 (BD Biosciences) double‐positive cells after extracellular staining. Following, the expression levels of IL‐4‐PerCP/Cy5.5, IL‐6‐FITC and IL‐13‐PE (BD Biosciences) in basophils were quantified and expressed as a positive percentage of total basophils. Isotype controls (mouse IgG1‐PE, mouse IgG1‐Alexa Fluor 647, mouse IgG1‐PE‐Cy7, mouse IgG1‐PerCP/Cy5.5, Rat IgG2a‐FITC and Rat IgG1‐PE) were used for each step.

A FACScanto™ II flow cytometer (Becton Dickinson) and the FlowJo 7.6 software were used to acquire and analyse the data, respectively. In addition, number of basophils in peripheral blood of some patients with MCD were also determined by routine blood tests with automated numeration technics (Beckman coulter COULTER^®^ LH 750 System) for linear correlation analysis between FCM tests and routine blood tests.

### 2.6 Effect of serum from patients with MCD on the activation of basophils

Blood was collected from healthy volunteers (n = 9) to negatively select basophils and separate the serum. Serum was then collected from nine patients with MCD. EasySep™ human basophil enrichment kit (Stemcell Technologies) was used to negatively purify human basophils to obtain a purity of greater than 90% using flow cytometry.

Nine portions of basophils from normal individuals were placed into RPMI 1640 medium (Gibco Laboratories) with serum from patients with MCD (20%) and IL‐3 (20 ng/mL; PeproTech), then cultivated for 24 hours. Flow cytometry was then used to detect changes in the MFIs of CD203c and CD62L on basophils. Using the same method, nine portions of basophils from normal people were placed into the culture medium with allogeneic serum from healthy volunteers (20%) and IL‐3 (20 ng/mL). Before FCM test, cell viability was tested by 0.2% trypan blue solution (Sigma).

### 2.7 Ethical approval

The study was approved by the ethics committee of the Affiliated Hospital of Guangdong Medical University (approval no. PJ2017054). The study conformed to the tenets of the Declaration of Helsinki. All patients provided informed consent before taking part in the study.

### 2.8 Statistical analysis

All statistical analysis was performed using SPSS 23.0 (SPSS, Inc). The normal distributed data were expressed as mean ± standard deviation, and two‐group comparisons were performed using paired or unpaired two‐tailed *t* test. Non‐normal distributed data were expressed as median (*P*_25_, *P*_75_), and two‐group comparisons were performed using Mann‐Whitney *U* test. Linear regression analysis was used to determine correlation between routine blood tests and flow cytometry tests of peripheral basophil counts. *P* < .05 was considered to indicate statistical significance.

## 3 RESULTS

### 3.1 Patients at the initial onset of MCD had increased counts and enhanced activity of peripheral basophils

**FIGURE 1:**
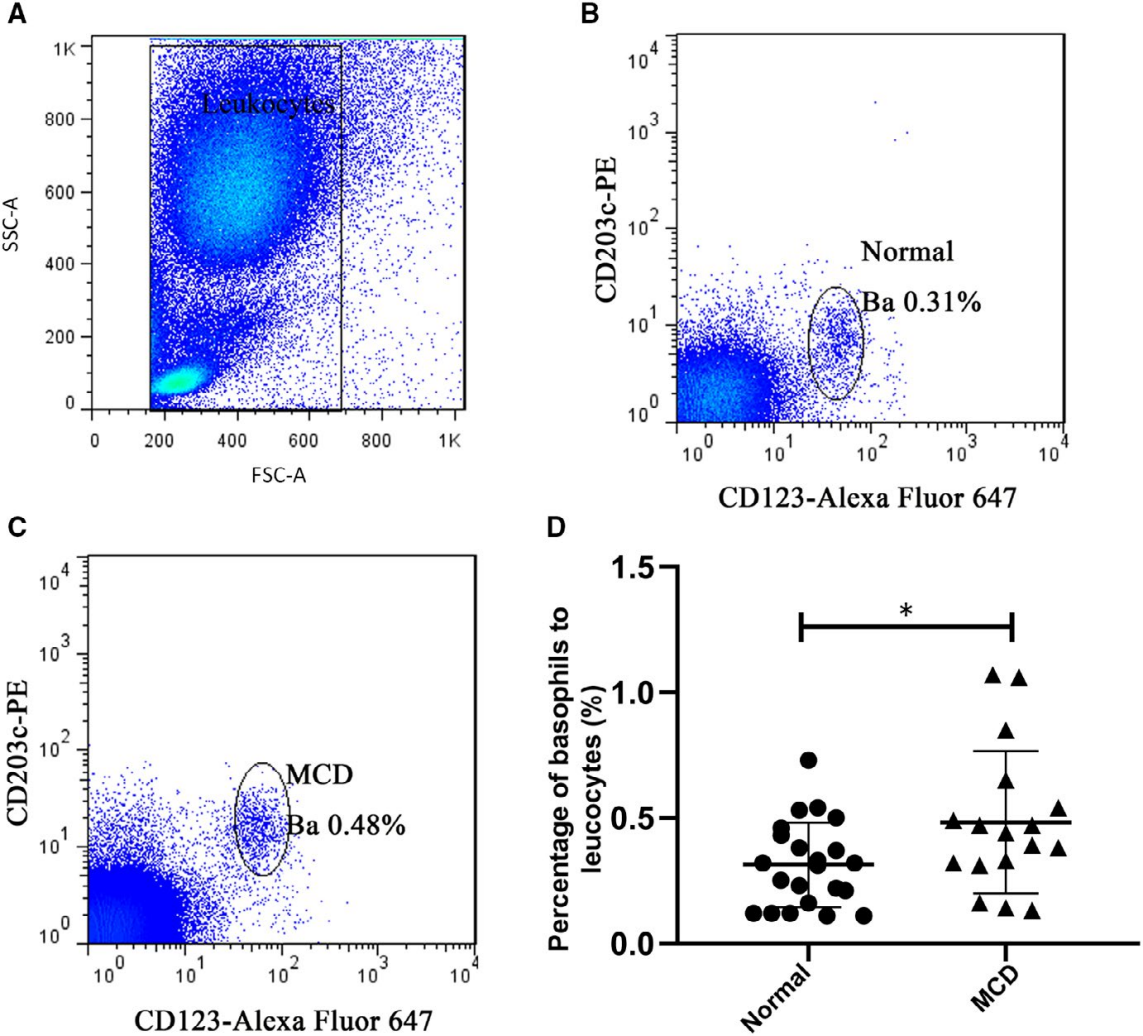
Ratios of peripheral basophils in white blood cells determined by flow cytometry. A, Representative flow cytometric scatter plot. The white blood cell group was used to set the gate to label white blood cells. B, C, Representative diagrams of basophil ratios in the normal control group and MCD group; basophils was defined as CD123^+^CD203c^+^. D, Comparison diagram of peripheral basophil ratios between the normal control group (n = 22) and the MCD group (n = 17). *P < .05, unpaired *t* test was performed. Ba, basophils

From March 2017 to February 2018, peripheral blood samples were collected from 17 patients at the initial onset of MCD (not yet treated) at our hospital. No statistical significance was found for age differences between patients and normal control groups (*P* > .05). Flow cytometry analysis demonstrated that the mean ratio of peripheral basophils in white blood cells (0.48% ± 0.29%) in the MCD group was significantly higher than that of the normal control group (0.31% ± 0.17%) (*P* < .05; Figure 1).

**FIGURE 2:**
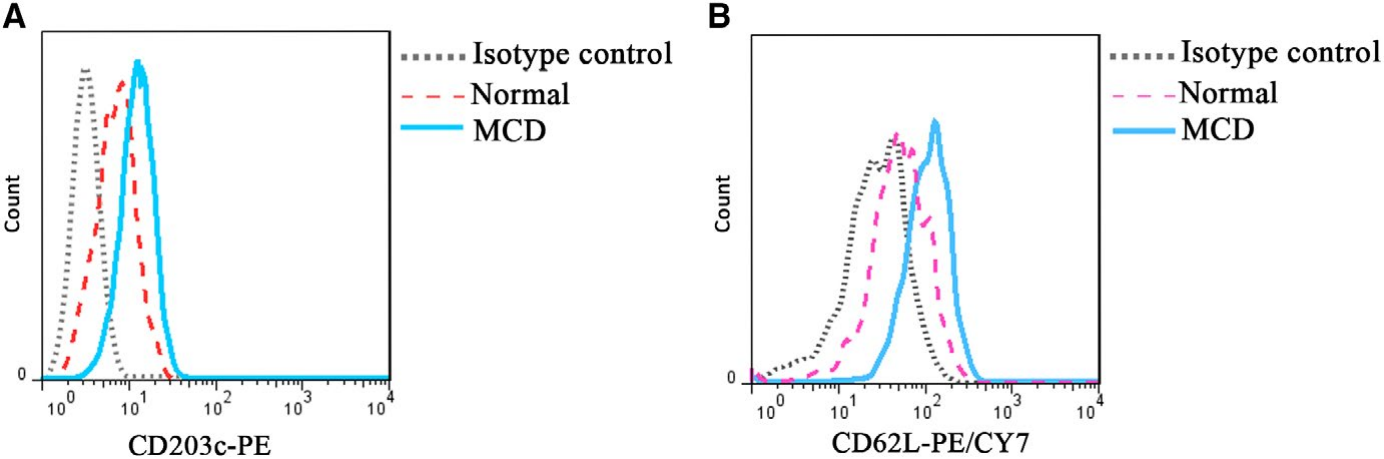
Representative diagram of peripheral basophil activation status in patients with MCD, as determined by flow cytometry. Representative diagram of MFI values of activation index CD203c (A) and CD62L (B) of basophils in patients with MCD and the normal control group. Ba, basophils; MFI, mean fluorescence intensity

During the same period under the same conditions, MFI values of CD203c and CD62L on basophils from patients with MCD were all higher than those of the normal control group (*P* < .05; Figure 2; Table 1).

**TABLE 1:** MFI values of basophil activation in the MCD and normal control groups in the same period

Activation index	MCD (n = 10)	Normal (n = 8)	*P*
CD203c MFI (mean ± SD)	14.8 ± 8.8	5.4 ± 2.2	.008
CD62L MFI (mean ± SD)	100.3 ± 61.6	35.8 ± 31.5	.016

Note

Unpaired *t* test was performed.

**FIGURE 3:**
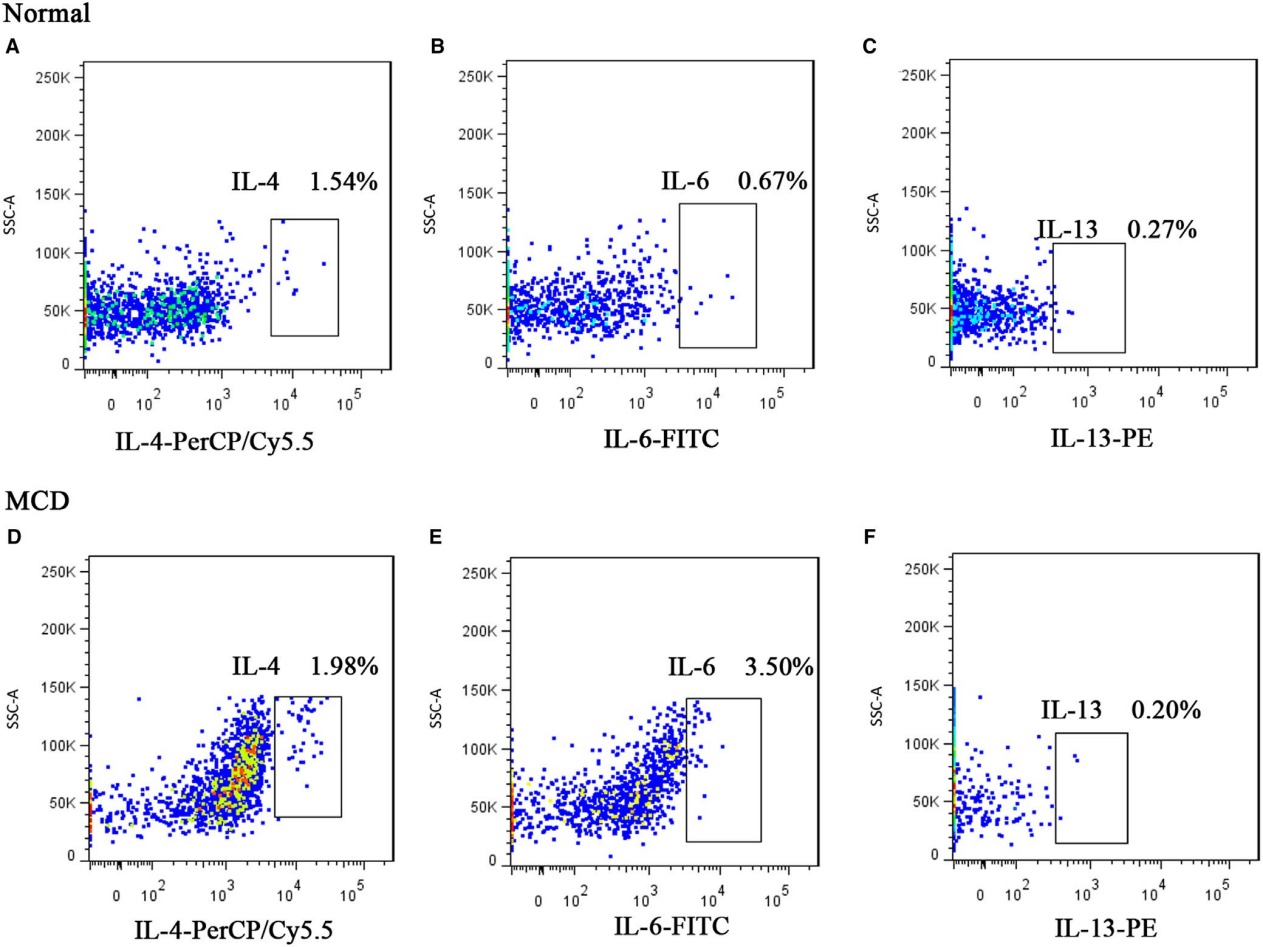
Representative diagram of the percentages of basophils with positive IL‐4, IL‐6 and IL‐13 to total basophils in patients with MCD and normal controls. A, B and C, Representative diagram of percentages of IL‐4^+^ basophils, IL‐6^+^ basophils and IL‐13^+^ basophils to total basophils, respectively, in normal controls. D, E, F, Representative diagram of percentages of IL‐4^+^ basophils, IL‐6^+^ basophils and IL‐13^+^ basophils to total basophils, respectively, in patients with initial onset of MCD. Ba, basophils

Flow cytometry further detected the expression of intracellular factors of basophils before treatment in patients with MCD. The percentages of IL‐4^+^ basophils and IL‐6^+^ basophils to total basophils in the MCD group (n = 15) were all higher than those of the normal control group (n = 16; *P* < .05). The percentage of IL‐13^+^ basophils was extremely low in the MCD group, and there was no significant difference compared with that of the normal control group (*P* > .05; Figure 3; Table 2).

**TABLE 2:** Comparison of percentage of intracellular factors‐positive basophils to the total basophils in the MCD and normal control groups

Intracellular factors	MCD (n = 15)	Normal (n = 16)	*P*
IL‐4^+^Ba M (*P*_25_, *P*_75_) %	1.98 (0.97, 4.67)	0.54 (0.38, 1.22)	.021
IL‐6^+^Ba M (*P*_25_, *P*_75_)%	3.5 (1.94, 6.03)	0.67 (0.32, 1.15)	.000
IL‐13^+^Ba M (*P*_25_, *P*_75_) %	0.20 (0.13, 2.09)	0.27 (0.08, 0.57)	.545

Note

Mann‐Whitney *U* test was performed.

Abbreviation: Ba, basophils.

### 3.2 Serum from patients with MCD activated basophils from healthy controls

After basophils from healthy controls were cultivated and stimulated in the culture medium with an allogeneic serum of normal individuals for 24 hours, the cell viability was all higher than 95%, and the MFI values of activated and labelled CD203c and CD62L showed no significant differences (*P* > .05). After cultivation and stimulation in the culture medium with serum from patients with MCD, the MFI values of CD203c and CD62L were all significantly increased (*P* < .05; Table 3).

**TABLE 3:** Changes in the activated parameters after basophils from normal individuals were stimulated and cultivated with serum from patients with MCD for 24 h

Activation index	Before stimulated (Normal, n = 9)	After stimulated (Normal, n = 9)	*P*	After stimulated (MCD, n = 9)	*P*
CD203c MFI	2930.3 ± 1247.5	2871.0 ± 1244.8	.439^a^	4182.6 ± 2401.5	.048^b^
CD62L MFI	5290.7 ± 2871.6	5720.0 ± 3242.5	.095^a^	9052.9 ± 3639.2	.038^b^

^a^ Before vs after stimulated with serum from normal individuals.

^b^ Before vs after stimulated with serum from patients with MCD; paired *t* test was performed.

### 3.3 Peripheral basophil counts assess MCD activity

**FIGURE 4:**
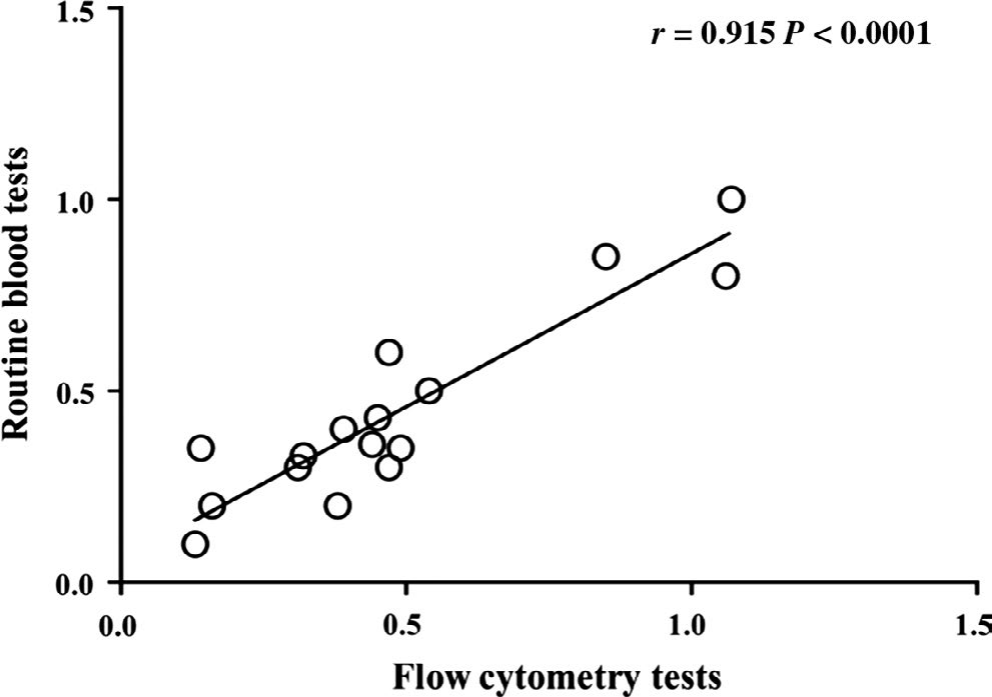
Linear correlation analysis of two methods for basophil identification. The test results between routine blood tests and flow cytometry tests of changes in peripheral basophil counts in healthy volunteers (n = 16)

Based on the above experimental findings, peripheral basophils from patients with MCD showed significantly increased quantity and enhanced activation. However, routine blood tests are used in clinical practice to obtain quantitative findings; therefore, we next used routine blood tests to evaluate the peripheral blood from the above‐mentioned 16 patients with MCD. There were no significant differences in the ratios of basophils, as determined by paired *t* tests (*P* = .731). Linear correlation analysis indicated that the two methods (FCM and routine blood tests) were significantly correlated (*r* = .915, *P* < .001; Figure 4). The above results indicated that the two methods were consistent regarding the detection of changes in basophil counts.

**FIGURE 5:**
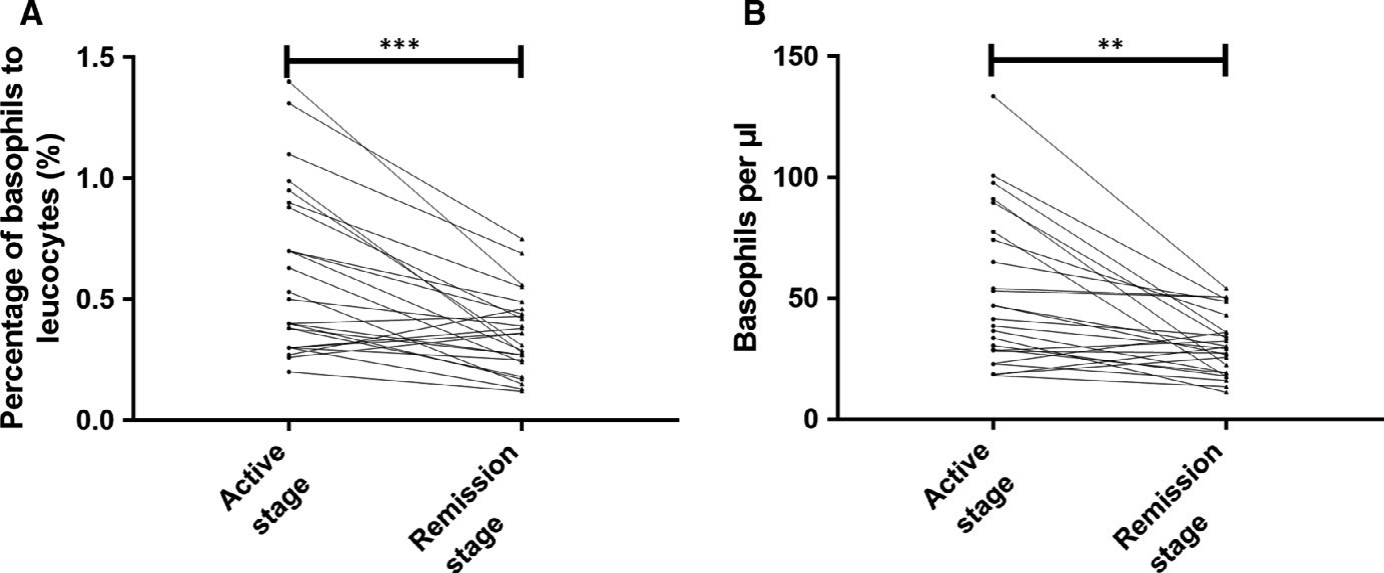
Changes in basophil ratios and counts during the active stage and remission stage in patients with MCD. A, Basophil ratios during the active stage and remission stage in patients with MCD (n = 25). B, Basophil counts in the active stage and remission stage in patients with MCD. ***P < .001; **P < .01, paired *t* test was performed

Next, we used retrospective analyses to investigate whether basophil count alone could be used to assess MCD activity. First, differences in peripheral basophil counts were investigated between the active and remission phases in patients with MCD. There were 25 cases of patients at the initial onset of MCD admitted into our hospital from 2015 to 2016, who had complete remission after a full course of treatment (>9 months). These patients had stopped glucocorticoid treatment for more than 1 month (allowing the influence of glucocorticoids on white blood cell counts and basophil counts to be excluded), and routine blood tests were then performed again to determine the ratio and count of basophils. The basophil ratio during the active stage (initial onset, before treatment) (0.61% ± 0.34%) was notably higher than the remission stage (0.36% ± 0.17%) (*P* < .001; Figure 5A). The basophil count in the active stage (51.9 ± 31.3/μL) was significantly higher than the remission stage (31.0 ± 12.7/μL) (*P* < .01; Figure 5B). Therefore, the basophil ratio and count during the active stage were higher than those during the remission stage in patients with MCD. These results suggested that peripheral basophil counts assess MCD activity.

### 3.4 Peripheral basophil counts could predict remission and relapse in patients with MCD

**FIGURE 6:**
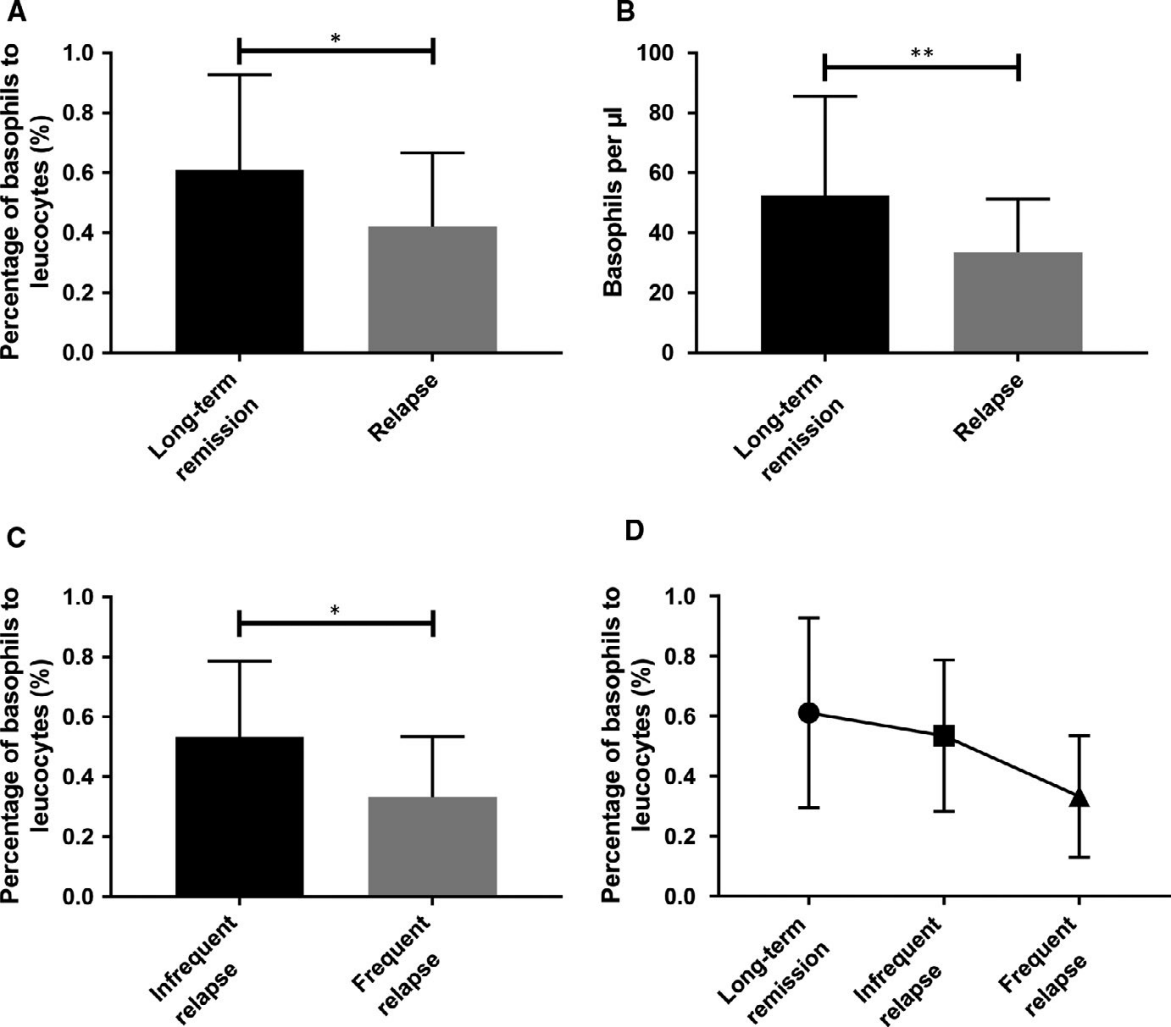
Comparison of basophil ratios and counts in patients with initial onset of MCD with different clinical outcomes. A, Comparison of basophil ratios during initial onset of MCD in the long‐term remission group (n = 33) and relapse group (n = 29). B, Comparison of basophil counts during initial onset of MCD in the long‐term remission group (n = 33) and relapse group (n = 29). C, Comparison of basophil ratios during initial onset of MCD in the infrequent relapse group (n = 13) and frequent relapse group (n = 16). D, Tendencies of basophil ratios in the long‐term remission group (n = 33), infrequent relapse group (n = 13) and frequent relapse group (n = 16). *P < .05; **P < .01, unpaired *t* test was performed

Another critical clinical issue is the peripheral basophil counts during remission and relapse in patients with MCD. There were 62 patients at the initial onset of MCD admitted from 2015 to 2016, who had negative urinary protein after the initial treatment and had follow‐ups for 1 year, including 33 cases of long‐term remission and 29 cases of relapse (16 cases of frequent relapse and 13 cases on infrequent relapse). The results indicated that the basophil ratio before treatment in the long‐term remission group (0.61% ± 0.32%, n = 33) was higher than the relapse group (0.42% ± 0.24%, n = 29) (*P* < .05; Figure 6A). The basophil count before the treatment in the long‐term remission group (52.5 ± 33.0/μL, n = 33) was also higher than the relapse group (34.7 ± 17.0/μL, n = 29; *P* < .01; Figure 6B). The basophil ratio before the treatment in the infrequent relapse group (0.53% ± 0.25%, n = 13) was higher than the frequent relapse group (0.33% ± 0.20%, n = 16) (*P* < .05; Figure 6C). Notably, the basophil count before treatment in the infrequent relapse group (38.6 ± 14.1/μL) was not significantly different compared with the frequent relapse group (31.5 ± 18.8/μL) (*P* = .276). The basophil ratios in the long‐term remission group, infrequent relapse group, and frequent relapse group showed gradual decreasing trends (Figure 6D). These results suggested that patients at the initial onset of MCD with a relatively high basophil ratio and count are more likely to have long‐term remission after treatment.

## 4 DISCUSSION

IgE and basophils maybe involved in the pathogenesis of MCD,^14^, ^18^ and Th cells may lead to the onset of MCD by up‐regulating Th2 cells, which express high levels of Th2 cytokines, including IL‐4 and IL‐13.^7^, ^8^, ^9^, ^10^, ^11^, ^12^, ^24^ Also, basophils are the dominant cell type producing Th2 cytokines in the early phase of Th2 response. In addition, IL‐4 originating from Th2 cells can act on B cells to produce IgE antibodies, which activate basophils to promote the pathogenic cycle.^25^, ^26^ The basophils in the peripheral blood white cells can be accurately detected using antibodies targeting CD123 and CD203c, two basophil‐sensitive activation indicators.^27^, ^28^, ^29^ Thus, activated basophils can directly affect the functions of T and B lymphocytes or indirectly affect T and B lymphocytes *via* generation of specific metabolites.

In our current study, we found that peripheral basophils in adult patients at the initial onset of MCD showed increased number and enhanced activity compared with that in patients with stable MCD. These findings were consistent with the results described by Pirotzky et al,^18^ further indicating that peripheral basophils in patients at the initial onset of MCD were activated and changes in the number and activation of basophils in patients with MCD were closely related to the activity of the disease.

Although the pathogenic factors associated with proteinuria in patients with MCD have not been defined, such pathogenic factors may originate from the blood circulation rather than the kidney.^16^, ^30^ When the serum of patients with MCD was used to treat basophils from healthy volunteers, basophils were activated, suggesting that the substance activating basophils may have originated from the serum, for example, IgE.

The biological functions of basophils are dependent on cell activation, which can be mediated by multiple stimulators, including IgE.^31^, ^32^ Serum IgE levels are increased in patients with MCD and then recovered to normal levels after remission.^14^, ^15^, ^33^ In contrast, pathological examination of the kidneys in patients with MCD showed no deposition of IgE or IgE‐circulating immune complexes (CIC), indicating that IgE was an intermediate factor in MCD onset. Therefore, activation of basophils may be mediated by IgE‐CIC or other IgE‐relevant compounds crosslinked with the high‐affinity receptor FcεRl of basophil IgE.^32^ Activated basophils exhibit up‐regulation of surface molecules, including CD203c and CD62L, and quickly produce cytokines, including IL‐4.^34^

The ratio of basophils positively expressing cytokines, including IL‐4, IL‐6, and IL‐13, is another parameter for the activation of peripheral basophils in patients with MCD. Compared with CD203c and CD62L, this ratio puts more emphasis on the functional expression of activated basophils, that is, whether the activated basophils can secrete active mediators. Researchers have concluded that basophils produce the essential cytokine IL‐4, which is required for the differentiation of Th2 cells, whereas IgE is involved in the main pathway mediating Th2‐cell activation.^35^ In our current study, we confirmed that the increased percentage of IL‐4‐positive basophils to total basophils in peripheral blood from patients with MCD was associated with activation of basophils and cytokine secretion in patients with MCD, thus highlighting the participation of these cells in the pathogenesis of MCD. IL‐6‐positive basophils in patients with MCD were also increased; however, no studies have demonstrated the roles of IL‐6 in the pathogenesis of MCD. Increased IL‐6 expression may only be a concomitant phenomenon of enhanced basophil activation and may not necessarily participate in the pathogenesis of MCD directly.

Several studies have demonstrated that serum IL‐13 is increased in patients with MCD during the active phase.^8^, ^36^ However, in this study, basophils expressed extremely low levels of IL‐13 in patients with MCD and healthy controls, suggesting that basophils may not act on podocytes by directly expressing IL‐13, but could release cytokines, including IL‐4, to mediate other cells (eg mediate Th0 cells to differentiate into Th2 cells) and produce IL‐13. Further studies are needed to determine whether higher concentrations of IL‐13 in the serum of patients with MCD are directly mediated by basophils.

Frequent relapse of MCD is a difficult problem to treat in clinical practice. Rituximab, an anti‐CD20 monoclonal antibody, can successfully treat frequently relapsed and refractory MCD by targeted clearance of CD20‐positive B lymphocytes,^37^ indicating that frequent relapse of MCD is closely related to the occurrence of B lymphocytes. IgE produced by B lymphocytes can mediate the activation of basophils. Accordingly, further studies are needed to evaluate the mechanisms through which basophils are involved in the relapse and remission of MCD.

The use of FCM to detect basophil activation involves complicated methods, which are difficult to carry out in clinical practice. In contrast, quantification can be achieved using routine blood cell analyzers; therefore, we investigated the relationships among basophil counts, MCD activity and clinical outcomes. The results indicated that changes in basophil counts determined by routine blood tests could be used to assess MCD activity in clinical practice; however, further studies are needed to confirm these findings. Another important factor is that the counts of peripheral basophils calculated by routine tests which are known not to be precise,^38^ which largely depend on the model of the machine whether or not have an independent basophil detection channel.

In our analysis of the relationships between basophils and MCD relapse, we incidentally found that after treatment with glucocorticoids, lower basophil count and ratio in patients at the initial onset of MCD before treatment were related to a higher possibility of relapse in the future. This phenomenon was difficult to reconcile with our understanding of basophils. Specifically, it was unclear why more basophils and more serious sensitization did not result in an easier relapse. According to current research on MCD,^39^ there are two possible explanations. One possibility is that basophils may be harmful during the pathogenesis of MCD. In patients with bronchial asthma,^40^, ^41^ with the same allergic reaction and frequent relapse as MCD, and in patients with autoimmune diseases, such as systemic lupus erythematosus,^42^, ^43^ basophils migrate from the blood circulation to the secondary lymphoid organs^44^ or site of inflammation during disease onset, resulting in decreased basophils in blood circulation and injury to migrated tissues. Unlike SLE, during the pathogenesis of MCD, there are no immune complexes or white blood cells deposition in the glomeruli. It is possible that no white blood cells migrate to the target organ during the pathogenesis. This may explain why MCD maintains high basophils at the onset, although this is only a speculation. Another possibility is that basophils may be beneficial during MCD onset. High levels of IL‐4 and IL‐13 in the serum of patients with MCD during the active phase are not directly or indirectly produced by basophils, and increased basophils in patients with MCD can inhibit the expression of pathogenic factors, thus resulting in long‐term remission in patients with higher peripheral basophil counts. Either way, further studies with larger sample sizes are needed to provide a reference for selecting an appropriate therapeutic regimen in clinical practice. Based on our findings, adult patients with lower basophil ratios before treatment may benefit from combining immunosuppressive agents to reduce relapse.

Certain inherent limitations were noted within the current study. One limitation of our study is that patients with non‐MCD nephrotic syndrome were not included as a control group, so changes and activation of basophils might not necessarily be unique in patients with nephrotic syndrome. Another is, there maybe have the presence of CD123^+^ pDCs in basophil gate due to lower expression of CD203c by basophils from normal controls, which may lead the real data for basophil frequency of normal control may slightly lower. Also, the results showed that compared with MCD patients, the frequency of basophils was significantly lower than MCD, even there maybe have the presence of pDCs in basophil gate of normal control. So, this interference did not affect the conclusion of this study.

These findings suggested that basophil may play a pathogenic role in adult‐onset MCD, and the increased number and activation of peripheral basophils could predict recurrence in adult MCD.

## CONFLICT OF INTEREST

The authors declare no conflict of interest.

## AUTHOR CONTRIBUTIONS

HH, YX and QP provided the idea and conceived and designed the experiments. HH, SL, XC and XL performed the experiments and analysed the data. HH, HX, SW and CY wrote the manuscript. HL supervised the study. All authors reviewed and approved the final manuscript.

## Data Availability

All data included in this study are available upon request by contact with the corresponding author.
